# Acute Kidney Injury Due to COVID-19 in Intensive Care Unit: An Analysis From a Latin-American Center

**DOI:** 10.3389/fmed.2021.620050

**Published:** 2021-06-04

**Authors:** Precil Diego Miranda de Menezes Neves, Victor Augusto Hamamoto Sato, Sara Mohrbacher, Bernadete Maria Coelho Ferreira, Érico Souza Oliveira, Leonardo Victor Barbosa Pereira, Alessandra Martins Bales, Luciana Loureiro Nardotto, Jéssica Nogueira Ferreira, David José Machado, Estêvão Bassi, Amilton Silva-Júnior, Pedro Renato Chocair, Américo Lourenço Cuvello-Neto

**Affiliations:** ^1^Nephrology and Dialysis Center, Oswaldo Cruz German Hospital, São Paulo, Brazil; ^2^Nephrology Division, University of São Paulo School of Medicine, São Paulo, Brazil; ^3^Intensive Care Unit, Oswaldo Cruz German Hospital, São Paulo, Brazil

**Keywords:** acute kidney injury, COVID-19, intensive care unit, SARS-CoV-2, dialysis, continuous renal replacement therapy

## Abstract

**Introduction:** The kidney may be affected by coronavirus (COVID-19) in the setting of acute kidney injury (AKI). Data about AKI in intensive care unit (ICU) patients in Latin America are scarce. We aimed to evaluate the risk of AKI, dialysis (HD), and death in ICU COVID-19 patients in a Brazilian center.

**Methods:** Analysis from medical records of COVID-19 patients in a Brazilian center.

**Results:** A total of 95 patients were analyzed. There was male predominance (64.2%), median age: 64.9 years, and previous history of hypertension and diabetes in 51.6 and 27.4%, respectively. AKI was diagnosed in 54 (56.8%) patients, and 32 (59.2%) of them required HD. Mortality rate was 17.9%. AKI patients when compared with no-AKI were more frequently hypertensive/diabetic and more often needed organ support therapies. Workups depicted more anemia, lymphopenia, and higher levels of inflammatory markers and higher mortality. Comparing patients who had undergone death to survivors, they were older, more frequently diabetic, and had worse SAPS3 and SOFA scores and need for organ support therapies, AKI, and HD. Multinomial logistic regression revealed that hypertension (*p* = 0.018) and mechanical ventilation (*p* = 0.002) were associated with AKI; hypertension (*p* = 0.002), mechanical ventilation (*p* = 0.008), and use of vasopressor (*p* = 0.027) to HD patients; and age >65 years (*p* = 0.03) and AKI (*p* = 0.04) were risk factors for death.

**Conclusions:** AKI was a common complication of ICU COVID-19 patients, and it was more frequent in patients with hypertension and need of organ support therapies. As well as age >65 years, AKI was an independent risk factor for death.

## Introduction

The first cases of atypical pneumonia of unidentified etiology were reported on December 30, 2019, in Wuhan, China. Severe acute respiratory syndrome coronavirus 2 (SARS-CoV-2) was identified by January 7, 2020, and the disease has since been named COVID-19. It was declared a pandemic in March 2020, affecting nearly every country. The initial clinical case series from China largely comprises hospitalized patients with severe pneumonia. Further data suggests that ~80% of the patients have a mild form of the disease, 20% require hospital admission, and ~5% require intensive care admission (1, 2).

The death rate from COVID-19 across different populations (3, 4) has its highest rate reported among older patients and those who are immunocompromised or have other comorbidities or lymphocytopenia (5, 6). Brazil is the most populated country in Latin America and was the first place to confirm a case on the continent on February 25, 2020, in the city of São Paulo (7). Since then, the number of people affected by the disease is growing, and Brazil has become, at the moment, the third country in the world in the number of total cases (8).

Acute kidney injury (AKI) is a syndrome. It is an important complication in patients admitted to hospitals (10–15% of all hospitalizations) (9) and in patients in the intensive care unit (ICU), where its prevalence can sometimes exceed 50% (10). The incidence of AKI in patients with COVID-19 depends on the profile of the analyzed patients, meaning their severity level and if they are in a ward, outpatient, or ICU environment. AKI during a hospital stay is reported with an average incidence of 11% (8–17%) overall with the highest ranges in the critically ill [23% (14–35%)] (11).

No data on AKI in Latin American countries has been published since the COVID-19 pandemic started. Thus, the purpose of this study is to assess the impact of AKI on the mortality and renal prognosis of patients with SARS-CoV-2 infection.

## Methodology

### Patient Selection

This is a retrospective, single-center study with the purpose of assessing the impact of AKI in patients with COVID-19. Adult patients hospitalized from February 28, 2020, to May 4, 2020, in the ICU of the Oswaldo Cruz German Hospital, Brazil, were chosen for the case study. All patients were diagnosed with COVID-19 infection based on reverse transcription polymerase chain reaction (RT-PCR) for the virus. Patients receiving palliative care or transferred to external services with no follow-up possibility were excluded from the case study.

### Sample Characterization

The patients were characterized for gender, age, and the presence of comorbidities (hypertension, diabetes mellitus, chronic obstructive pulmonary disease, chronic kidney disease, presence of active neoplasm, and immunosuppression use). The following complementary tests were analyzed: maximum D-dimer, C-reactive protein (CRP), lactate dehydrogenase (LDH), and blood count. We assessed the tests made on the day the nephrologist requested them for patients who had AKI, and we assessed the admission tests for those who did not have AKI. The organic dysfunctions assessed were mechanical ventilation required, use of extracorporeal membrane oxygenation (ECMO), vasopressor use, and renal replacement therapy required. AKI was defined as per the KDIGO (12) criteria based on the creatinine values and diuretic rhythm. AKI KDIGO 1 was defined as an increase of creatinine ≥0.3 mg/dl or 1.5–1.9 times baseline and/or urine output <0.5 ml/kg/h for 6–12 h. AKI KDIGO 2 was defined as an increase of creatinine of 2.0–2.9 times baseline and/or urine output <0.5 ml/kg/h for 12 h. AKI KDIGO 3 was defined as an increase of creatinine of 3.0 times baseline or increase in serum creatinine to ≥4.0 mg/dl or initiation of renal replacement therapy and/or urine output <0.3 ml/kg/h for ≥24 h or anuria for ≥12 h. The instituted treatments were also analyzed (use of antibiotics, anti-IL6, ivermectin, and nitazoxanide). The reference values for the lab tests are as follows: D-dimer: <500 ng/mL FEU, CRP: <1 mg/dL, LDH: 240 to 480 U/L. The following variables were analyzed: length of ICU stay, mechanical ventilation time, and total hospitalization period. Because many patients received a tracheotomy and the continuous use of CPAP/BIPAP was required, we considered the moment in which the patient no longer needed continuous CPAP/BIPAP support as the end of mechanical ventilation time.

### Hemodialysis Protocol

The hemodialysis initiation timing and modality were defined by the nephrology team. We placed a hemodialysis catheter of 15.5 Fr in all patients, and the puncture site was chosen according to each patient's clinical setting. Our protocol indicates continuous venous–venous hemodiafiltration (CVVHDF) as the method of choice for those patients who required vasoactive drug infusion and/or presented evidence of hypervolemia. CVVHDF was performed using the Prismaflex system (Baxter, IL, USA) and AN69 ST150 (Baxter, IL, USA) as well as Oxiris® (Baxter, IL, USA) dialysis filters. The prescribed dialysis dose was 25–30 mL/kg/h of effluent and with regional citrate anticoagulation. Slow low-efficiency dialysis and classic hemodialysis were performed using a 4008S hemodialysis system (Fresenius, Germany) for patients with no need of vasoactive drugs.

### Endpoint Analysis

The primary endpoint assessed was death attributed to AKI and dialysis need; the secondary endpoints were risk factors for AKI development, dialysis required, and death during the hospital stay.

### Statistical Analysis

The distribution of variables was assessed with the Shapiro–Wilk test. Qualitative variables were expressed as proportions and compared against each other via the chi-squared test or Fisher's exact test. Variables following a parametric distribution were expressed as mean values ± standard error and compared against each other with Student's *t*-test. Variables with non-parametric distributions were expressed as median values (p25 and p75) and compared against each other with the Mann–Whitney *U*-test. Logistic regression was used in multivariate analysis. Statistical significance was attributed to *p* < 0.05. We used an ROC curve to analyze the age difference between patients who died and survived.

### Ethical Aspects

The current study is a subanalysis of the *Diagnóstico e prática cl*í*nica em s*í*ndromes gripais agudas durante a pandemia por SARS-COV2 em pacientes internados em unidades de baixa e alta complexidade hospitalar* (Diagnosis and clinical practice in acute flu syndromes during the SARS-COV2 pandemic in patients hospitalized in low and high hospital complexity units) project, approved by the Brazilian Ethics Committee under number 30264920.6.1001.0070.

## Results

From an initial sample of 102 patients, seven were excluded after receiving palliative care or being transferred to other centers throughout the hospital stay. Thus, 95 patients remained for the final data analysis. The baseline characteristics of the patients are outlined in Table 1. Most of them were older adult patients (64.9 ± 15.1 years of age) and men (64.2%), and diabetes and hypertension were the frequent comorbidities (51.6 and 27.4% of the patients, respectively). In addition, 11 (11.6%) patients had immunosuppression status with most of them (54%) being on chemotherapy treatment for an active cancer. Chronic kidney disease was present in 16.8% of the patients, three patients had CKD stage 2 (18.8%), seven had CKD stage 3 (43.8%), and six had CKD stage IV (37.4%). Regarding organic support therapies, the same proportion of patients was receiving mechanical ventilation and using vasopressors (54/56.8%), and three (3.7%) were submitted to ECMO therapy. AKI was diagnosed in 54 (56.8%) of the cases with 12 (22.2%) being KDIGO 1, four (7.4%) KDIGO 2, and 38 (70.3%) KDIGO 3. From the patients with AKI KDIGO 3, 32 (84.2%) required dialysis therapy, and the others continued with conservative treatment due to not presenting oliguria or hydroelectrolytic disorder nor acid-base indicating renal function replacement therapy. The average follow-up of the patients was 24 ± 9.3 days, and when completed, 17 (17.9%) patients died, 62 (65.3%) were discharged from the hospital, and 16 (16.8%) remained hospitalized. Analysis of urine spot was available in 40 patients, of which 21 (52%) had hematuria and 20 (50%) had proteinuria >300 mg/g. Hematuria was more frequent in patients who developed AKI (80.9 vs. 19%, *p* = 0.02), but similar results were not observed for proteinuria (56 vs. 25%, *p* = 0.11).

**Table 1:** Comparative clinical data, laboratory tests, and treatment in patients who presented with acute kidney injury and need of dialysis.

	**Overall (*n* = 95)**	**AKI (*n* = 54)**	**No AKI (*n* = 41)**	***p***	**Dialysis (*n* = 32)**	**No dialysis (*n* = 63)**	***p***
Age (years)	64.9 ± 15.1	66.8 ± 14.5	62.5 ± 15.8	0.175	68.8 ± 12.5	62.9 ± 16.1	0.76
Male (*n*/%)	61/64.2	35/64.8	26/63.4	0.88	11/34.4	23/36.5	0.838
Hypertension (*n*/%)	49/51.6	37/68.5	12/29.3	<0.001	26/81.2	23/36.5	<0.001
Diabetes (*n*/%)	26/27.4	22/40.7	4/9.8	0.001	13/40.6	13/20.6	0.039
COPD/Asthma (*n*/%)	14/14.8	9/16.7	5/12.2	0.543	3/9.4	11/17.5	0.293
Chronic kidney disease (*n*/%)	16/16.8	10/18.5	6/14.6	0.616	6/18.8	10/15.9	0.723
Obesity (*n*/%)	29/30.5	17/31.5	12/29.3	0.50	11/34.4	18/28.6	0.67
Cancer (*n*/%)	4/4.2	3/5.6	1/2.4	0.454	0/0	4/6.3	0.145
Immunosuppression (*n*/%)	11/11.6	9/16.7	2/4.9	0.07	7/21.9	4/6.3	0.02
Body Mass Index (kg/m^2^)	27.8 (24.7–30.4)	28.5 (24.8–30.8)	26.7 (24.6–30.5)	0.388	28.3 (25–31.5)	27 (24.4–30.4)	0.508
SAPS3	48 ± 14.1	52.4 ± 15	44.2 ± 11.6	0.005	54 ± 13.9	46.2 ± 13.7	0.01
SOFA score	3.25 (2–6)	4.5 (3–7.25)	3 (1–3.75)	<0.001	5.5 (3–8)	3 (1–5)	<0.001
Mechanical ventilation (*n*/%)	54/56.8	43/79.6	11/26.8	<0.001	30/93.8	24/38.1	<0.001
ECMO (*n*/%)	3/3.7	3/5.6	0/0	0.125	2/6.2	1/1.6	0.219
Vasopressor (*n*/%)	54/56.8	42/77.8	12/22.2	<0.001	32/100	22/34.9	<0.001
**Laboratory tests**							
Hemoglobin (g/dL)	13 (11–14)	12 (10–14)	14 (12–14)	0.005	12 (10–13.7)	13 (12–14)	0.004
Leucocytes (×103/*mm*^3^)	6,760 (4,940–9,927)	9,100 (5,740–12,000)	5,900 (4,670–6,775)	<0.001	9,860 (6,869–14,005)	6,085 (4,675–8,450)	<0.001
Lymphocytes (×103/*mm*3)	865 (600–1,237)	750 (500–1,181)	1,020 (790–1,257)	0.003	730 (512–1,175)	950 (706–1,256)	0.006
Platelets (×103/*mm*3)	197 (145–257)	223 (168–305)	166 (137–237)	0.009	241 (169–312)	179 (143–245)	0.027
D-Dimer (ng/mL FEU)	2148.5 (1,064–7421.2)	3285.5 (1435.5–10,000)	1,285 (823.7–3648.2)	0.004	3,806 (1,708–10,000)	1,472 (837–3,942)	0.005
Reactive C-protein (mg/dl)	25 (15–34)	31.9 ± 12.2	17.4 ± 11.7	<0.001	34.8 ± 13.7	21. ±11.5	<0.001
LDH (U/L)	739 (574–964)	742 (623.5–1032.5)	738 (512.2–865.5)	0.208	36.5 (623.2–1126.5)	712 (564–875)	0.039
**Treatment (*****n*****/%)**							
Antibiotic	89/93.7	54/100	35/85.4	0.004	32/100	57/90.5	0.07
Anti-IL6	4/4.2	2/3.7	2/4.9	0.778	2/6.2	2/3.2	0.481
Nitazoxanide	4/4.2	2/3.7	2/4.9	0.778	2/6.2	2/3.2	0.481
Ivermectin	5/5.3	2/3.7	3/7.3	0.435	2/6.2	3/4.8	0.759
Length of mechanical ventilation (days)	17.5 (10–28)	15 (10–27)	11 (8–18)	0.19	19 (10–27)	12 (8–23)	0.157
ICU stay (days)	19 (10.8–25.8)	18 (13–26)	3 (1–10)	<0.001	23 (17–27)	8 (2–16)	<0.001
Length of stay (days)	32 ± 16.8	34.1 ± 14.1	14.8 ± 11.1	<0.001	40.4 ± 13.6	18.6 ± 12.3	<0.001
Death (*n*/%)	17/17.9	15/27.8	2/4.9	0.004	11/34.4	6/9.5	0.003

Patients who developed AKI were more frequently hypertensive (68.5 vs. 29.3%; *p* < 0.001) and diabetic (40.7 vs. 9.8%; *p* = 0.001) and received mechanical ventilation (79.6 vs. 26.8%; *p* < 0.001) and vasoactive drugs (77.8 vs. 22.2%; *p* < 0.001) more frequently when compared with those that did not (Table 1), therefore reflecting a higher severity that was confirmed through the worst SAPS3 (52.4 vs. 44.2; *p* = 0.005) and SOFA (4.5 vs. 3; *p* < 0.001) scores. Regarding their lab tests, they had the lowest hemoglobin results (12 vs. 14 g/dL; *p* = 0.005), the highest lymphopenia results (750 vs. 1,020/mm^3^; *p* = 0.003), and the highest D-dimer (3285.5 vs. 1,285 ng/mL FEU; *p* = 0.004) and CRP (31.9 vs. 17.4 mg/dL; *p* < 0.001) values. The adopted therapies included receiving antibiotics (100 vs. 85.4%; *p* = 0.004) more frequently. Being diagnosed with AKI increased the ICU stay (18 vs. 3 days; *p* < 0.001), hospital stay (34.1 vs. 14.8 days; *p* < 0.001), and increased the mortality rates (27.8 vs. 4.9%; *p* = 0.004).

Patients requiring hemodialysis were more frequently hypertensive (81.2 vs. 36.5%; *p* < 0.001), diabetic (40.6 vs. 20.6%; *p* = 0.001), and immunosuppressed (21.9 vs. 6.3%; *p* = 0.02) when compared with those who were not submitted to the procedure (Table 1). There was a higher indication rate of mechanical ventilation in patients under dialytic treatment (93.8 vs. 38.1%; *p* < 0.001), using vasoactive drugs (100 vs. 34.9%; *p* < 0.001), and with the worst SAPS3 (54 vs. 46.2; *p* = 0.01) and SOFA (5.5 vs. 3; *p* < 0.001) scores. They also had the lowest hemoglobin results (12 vs. 13 g/dL; *p* = 0.004), the highest lymphopenia results (730 vs. 950/mm^3^; *p* = 0.006), and the highest D-dimer (3,806 vs. 1,472 ng/mL FEU; *p* = 0.005) and CRP (34.8 vs. 21 mg/dL; *p* < 0.001) values. When hemodialysis was required, it increased the ICU stay (23 vs. 8 days; *p* < 0.001) and hospital stay (40.4 vs. 18.6 days; *p* < 0.001) and also increased the mortality rates (34.4 vs. 9.5%; *p* = 0.003).

From the 32 patients submitted to dialytic therapy, 26 (81.3%) underwent continuous renal replacement therapy and six (18.7 %) received intermittent hemodialysis with the average dialysis time being 11 ± 7 days. Furthermore, 11 (34.4%) of the patients receiving dialysis died, and 13 (40.62%) from the remaining 21 (65.6%) experienced renal function recovery and maintained an estimate glomerular filtration rate using CKD-EPI = 23 (18–50 ml/min/1.73 m^2^) until hospital discharge, and another 8 (25.6%) remained dialysis-dependent.

When we analyzed patients who died and compared them with those who were still alive (Table 2), those who died were older (78.6 vs. 61.9 years of age; *p* = 0.04), more frequently diabetic (47.1 vs. 23.1%; *p* = 0.04), immunosuppressed (29.4 vs. 7.7%; *p* = 0.01), and received mechanical ventilation (88.8 vs. 72.2%; *p* < 0.004), and vasoactive drugs (94.1 vs. 48.7%; *p* = 0.001) more frequently; they also developed AKI (88.2 vs. 50%; *p* = 0.004), and hemodialysis was required (54.7 vs. 26.9%; *p* = 0.003) more frequently; therefore reflecting the worst SAPS3 (62.8 vs. 45.8; *p* < 0.001) and SOFA (8 vs. 3; *p* < 0.001) scores, respectively. They had the lowest hemoglobin results (11.8 vs. 13 g/dL; *p* = 0.01), the highest lymphopenia results (650 vs. 940/mm^3^; *p* = 0.01), and the highest CRP (31.9 vs. 24 mg/dL; *p* < 0.02) values. There were no differences regarding the adopted therapies.

**Table 2:** Comparative clinical data, laboratory tests, and treatment in patients who had undergone death and survivors.

	**Death (*n* = 17)**	**Survivors (*n* = 78)**	***p***
Age (years)	78.6 ± 8.6	61.9 ±14.6	0.04
Male (*n*/%)	9/52.9	52/66.7	0.285
Hypertension (*n*/%)	12/70.6	37/47.4	0.08
Diabetes (*n*/%)	8/47.1	18/23.1	0.04
COPD/Asthma (*n*/%)	4/23.5	10/12.8	0.259
Chronic kidney disease (*n*/%)	4/23.5	12/15.4	0.416
Obesity (*n*/%)	6/35.3	23/29.9	0.661
Cancer (*n*/%)	2/11.8	2/2.6	0.087
Immunosuppression (*n*/%)	5/29.4	6/7.7	0.011
Body Mass Index (kg/m^2^)	27.9 (21.8–30.1)	28 (25–31.9)	0.661
SAPS3	62.8 ± 14.3	45.8 ± 12.2	<0.001
SOFA	8 (5.5–9)	3 (2–5)	<0.001
Mechanical ventilation (*n*/%)	15/88.2	39/72.2	0.004
ECMO (*n*/%)	0/0	3/3.8	0.411
Vasopressor (*n*/%)	16/94.1	38/48.7	0.001
Acute kidney injury (*n*/%)	15/88.2	39/50	0.004
Dialysis (*n*/%)	11/64.7	21/26.9	0.003
**Laboratory tests**			
Hemoglobin (g/dL)	11.8 (9.4–12.7)	13 (11.2–14.1)	0.017
Leucocytes (×10^3^/mm^3^)	9,100 (5,170–14,815)	6,740 (4,930–9,540)	0.209
Lymphocytes (×10^3^/mm^3^)	650 (425–950)	940 (677.5–1,257)	0.014
Platelets (×10^3^/mm^3^)	151 (80–201)	145 (125–196)	0.848
D-Dimer (ng/mL FEU)	3,280 (1,676–10,000)	1,790 (970–5,814)	0.290
Reactive C-protein (mg/dl)	31.9 (22.8–38.9)	24 (14.5–34)	0.025
LDH (U/L)	809 (574.3–1,133)	737 (571–893)	0.327
**Treatment (*****n*****/%)**			
Antibiotic	17/100	72/92.3	0.237
Anti-IL6	0/0	4/5.1	0.340
Nitazoxanide	0/0	4/5.1	0.340
Ivermectin	0/0	5/6.4	0.283

*COPD, chronic obstructive pulmonary disease; ECMO, extracorporeal membrane of oxygenation; ICU, Intensive Care Unit; LDH, lactate dehydrogenase*.

In the multivariate multinomial logistic regression analysis (Table 3), it was predicted that patients who developed AKI more commonly had a previous diagnosis of hypertension (OR: 3.89; CI: 1.26–12.05; *p* = 0.018) and need of mechanical ventilation (OR: 11.54; CI: 2.42–55.14; *p* = 0.002); in addition, hypertension (OR: 11.14; CI: 2.43–51.11; *p* = 0.002), mechanical ventilation (OR: 12.79; CI: 1.92–85.20; *p* = 0.008), and the use of vasopressor (OR: 8.62; CI: 1.28–57.96; *p* = 0.027) were more frequent in patients who required hemodialysis therapy. Age >65 years (OR: 3.92; CI: 1.12–13.76; *p* = 0.03) and AKI (OR: 5.74; CI: 1.08–30.6; *p* = 0.04) were independent risk factors for death.

**Table 3:** Multinomial logistic regression predicting the risk factors for AKI, dialysis and death.

**Variable**	**OR**	**CI (95%)**	***p***
**Acute kidney injury**			
Hypertension	3.89	1.26–12.05	0.018
Diabetes	2.67	0.63–11.28	0.182
Mechanical ventilation	11.54	2.42–55.14	0.002
Vasopressor	0.52	0.11–2.59	0.426
Antibiotics	8.13	0.56–117.33	0.124
**Dialysis**			
Hypertension	11.14	2.43–51.11	0.002
Diabetes	0.68	0.07–1.41	0.129
Mechanical ventilation	12.79	1.92–85.20	0.008
Vasopressor	8.62	1.28–57.96	0.027
**Death**			
Age >65 years	3.92	1.12–13.76	0.033
Diabetes	1.24	0.37–4.23	0.726
Mechanical ventilation	1.25	0.14–11	0.840
Vasopressor	7.12	0.54–94.57	0.137
Acute kidney injury	5.74	1.08–30.6	0.041

*OR, odds ratio; CI, confidence interval*.

## Discussion

Several kidney problems associated with COVID have been previously described. The main problem is in the acute tubular injury spectrum (11, 12). Moreover, collapsing glomerulopathy (13–19) and thrombotic microangiopathy (20) cases have been described in the glomerulopathy spectrum. Other issues that have already been described are renal infarction (21, 22), acute pyelonephritis, rhabdomyolysis, and lymphocytic infiltrates (23).

It is claimed that renal injury mechanisms associated with COVID-19 involve inflammatory phenomena, crosstalk with other organs concurrently affected, and complications associated with organic dysfunction treatments. A phenomenon called a “cytokine storm” is characterized as a massive inflammatory cytokine release, mainly IL-6, therefore resulting in systemic vasodilation with increased vascular and endothelial permeability, which may ultimately lead to AKI and shock. Endothelial injury can also lead to dysfunctions in different organs, such as the heart and lungs, muscle injury, and rhabdomyolysis, in turn resulting in renal injury through an organic crosstalking mechanism. Furthermore, hypervolemia, loss of fluid to the third space, and endotoxin release are the last systemic complications described that contribute to AKI associated with viral conditions (24, 25).

The mechanisms of renal injury during COVID-19 are difficult to study due to the interference of several coexisting factors, such as polypharmacy, hypoxia, and cytokine storm. Just as in severe acute respiratory syndrome coronavirus, SARS-CoV-2 virus entry into target cells is facilitated by the presence of angiotensin-converting enzyme 2 (ACE2) expressed in respiratory cells as well as renal tubular cells and podocytes, making the hypothesis of a direct renal infection particularly intriguing (26).

Interestingly, viral nephropathies with direct tubular infection and viral replication within epithelial cells (i.e., BK virus nephropathy) are usually associated with substantial viruria due to the direct release of viral particles in the tubular lumen following cytopathic cell injury. Thus far, there is no solid evidence of a correlation between urinary levels of SARS-CoV-2 viral copies and the degree of kidney injury in patients with COVID-19 (27). Moreover, the presence of viral particles by pure morphologic evaluation (EM) does not by itself demonstrate a direct cytopathic effect nor a replicative potential because it only supports viral entry. More specific morphologic correlates of viral cytopathy (nuclear atypia/dysmorphology and syncytia) have not yet been shown.

A study analyzing the transcriptomics of renal cells in patients with COVID-19 (28) also demonstrates that the cytopathic virus effect may occur directly in proximal tubular cells and podocytes, therefore contributing to the onset of the disease through hyperexpression and recruitment of angiotensin-converting enzyme 2 (ACE2) and cellular transmembrane serine proteases (TMPRSSs), which are crucial for the virus internalization in affected cells.

In our study, the impact of AKI in the prognosis of critically ill patients with COVID is expressive. This finding has already been reported in a Chinese case study published by Cheng et al. (29) and in data from a retrospective study by 13 hospitals in the United States (30). The required renal replacement therapy results in poorer outcomes for these patients. Dialysis caused an almost ten-fold mortality increase when compared with patients without AKI in both studies. In our study, the presence of comorbidities (such as systemic hypertension and diabetes mellitus) was correlated with renal injury, therefore depicting the poor, preexisting conditions of these patients. In addition, the required mechanical ventilation and the use of vasopressor support in our data were important for the development of renal injury, thus expressing the serious conditions of the patients.

Another analysis of 3,099 ICU patients diagnosed with COVID-19 from 67 hospitals in the United States (31) revealed that 637 (20.6%) patients required dialysis. The mortality rate of those who needed renal replacement therapy was 63.3 and 33.8% were discharged from hospital still requiring dialysis. Moreover, in a follow-up 60 days after ICU admission, 18.1% of discharged patients were still maintained on regular dialysis.

In an English multicentric series (32) of 1,032 inpatients, of which 165 were in the ICU, the incidence of AKI in critical patients was 44.2% with a higher mortality in patients with AKI compared with non-AKI patients (52.1 vs. 22.8, *p* < 0.001) with similar results observed in French patients (33). In a Brazilian single-center study (34) with 201 ICU patients, 101 (50.2%) developed AKI and 32 (17%) required dialysis. The overall mortality was 14.4%, being higher in the AKI group of patients.

Risk factors for AKI in critical patients with COVID-19 vary according to the population. Older age, male gender, black race, obesity, diabetes, hypertension, heart failure, chronic kidney disease, leukocytosis, anemia, lymphopenia, increase in levels of serum inflammatory markers (D-dimer, PCR, and IL-6), and need for mechanical ventilation and vasoactive drugs as well as the use of angiotensin-converting enzyme inhibitors were associated with a higher risk of AKI development in COVID-19 critical patients (30–40). Herein, chronic kidney disease was more frequent in patients who developed AKI (18.5 vs. 14.6%), however with no statistical significance. This finding may be explained by the small number of patients with previous CKD diagnosis (16 patients) in addition to the fact that the diagnosis of CKD may reflect changes in renal function and/or evidence of structural changes with preserved renal function (41). Another interesting fact from our cohort is that some patients who developed AKI (15.3%), even reaching KDIGO 3 by the creatinine increase criteria, did not require renal replacement therapy because they did not present oliguria/anuria and some even polyuria. Due to the preserved diuresis, such patients did not develop uremia, hypervolemia, or other hydroelectrolytic disorders that required dialysis treatment. Regarding risk factors for mortality in COVID-19 patients, another case series (29–31, 34) found a correlation between obesity and mortality. This finding was not evidenced in our cohort; however, only 30.5% of the patients had an obesity diagnosis with most of them having a BMI in the range from 24.7 to 30.4 kg/m^2^ (p25; p75), differently from those from other series in which the obesity percentage and the BMI values were both higher.

Our study has some limitations, including being performed in a single center, and our data was collected from an electronic chart analysis from fewer than 100 critical patients; however, such factors resulted in standardized actions being taken and information registered in charts. Another limitation is the fact that it was performed in a private hospital; however, private hospitals were the first to receive patients with COVID-19 in Brazil.

Our study brings AKI data for critically ill patients with COVID-19 in a Brazilian center. AKI is a frequent finding, occurring in more than half of the patients, and dialysis is required in one third of the total case study. Previous comorbidities required organic systemic therapy use, lymphopenia, and elevated D-dimer, and CRP levels were associated with increased AKI development risk. Greater mortality rates were observed in older adult patients, and AKI was an independent risk factor for death. Therefore, we have exposed AKI data in critically ill patients with COVID-19 in a case study in Latin America, for which there is scarce data provided until now.

## Data Availability Statement

The original contributions presented in the study are included in the article/supplementary material, further inquiries can be directed to the corresponding author/s.

## Ethics Statement

The studies involving human participants were reviewed and approved by Brazilian Ethics Committee, number 30264920.6.1001.0070. Written informed consent for participation was not required for this study in accordance with the national legislation and the institutional requirements.

## Author Contributions

PN, VS, and PC: conception or design, analysis and interpretation of data, drafting the article, providing intellectual content, and final approval of the version. SM, ÉO, LP, AB, LN, JF, DM, EB, and AS-J: conception or design, providing intellectual content, and final approval of the version. BF: drafting the article, providing intellectual content, and final approval of the version. AC-N: conception or design, drafting the article, analysis and interpretation of data, providing intellectual content, and final approval of the version. All authors contributed to the article and approved the submitted version.

## Conflict of Interest

The authors declare that the research was conducted in the absence of any commercial or financial relationships that could be construed as a potential conflict of interest.
